# Molecular polymerization strategy for stable perovskite solar cells with low lead leakage

**DOI:** 10.1126/sciadv.ado7318

**Published:** 2025-05-07

**Authors:** Qixin Zhuang, Zhiyuan Xu, Haiyun Li, Cong Zhang, Cheng Gong, Huaxin Wang, Xiong Li, Zhigang Zang

**Affiliations:** ^1^ College of Optoelectronic Engineering, Chongqing University, Chongqing 400044, China.; ^2^ Michael Grätzel Center for Mesoscopic Solar Cells, Wuhan National Laboratory for Optoelectronics, Huazhong University of Science and Technology, Wuhan, China.; ^3^ School of Information Science and Engineering, Yanshan University, Qinhuangdao 066004, China.; ^4^ Henan Zhitong Optoelectronics Co., Ltd, Xuchang 461000, China.

## Abstract

Lead leakage and stability are the main challenges for the commercialization of perovskite solar cells (PSCs). Here, we propose adding *N*,*N*′-bis(acryloyl)cystamine (BAC) to the perovskite precursor solution, which facilitates the formation of polymer BAC (PBAC) at the grain boundaries during the annealing process of films. The PBAC can effectively passivate the defects and reduce the risk of lead leakage. Consequently, the PBAC-modified PSCs achieve an efficiency of 25.53% (0.1 square centimeters) (certified efficiency of 25.24%) and 24.03% (1.0 square centimeters). Moreover, after 1500 hours of continuous maximum power point tracking under simulated AM 1.5 illumination and 2000 hours of exposure to damp heat conditions (85°C and 85% relative humidity), the device retains approximately 96 and 81% of its initial power conversion efficiency, respectively. In addition, PBAC can effectively reduce lead leakage by nearly 72% by immersing the PSCs in water for 480 minutes.

## Teaser

The polymer PBAC formed at the perovskite grain boundaries can enhance the device efficiency and stability with low lead leakage.

## INTRODUCTION

Perovskite solar cells (PSCs) have gained increasing favor in both academic and industrial communities in recent years because of their solution processability and exceptional photovoltaic properties. Now, the certified power conversion efficiency (PCE) of PSCs has surpassed 26% (*1*–*3*). However, practical applications of PSCs still face challenges related to their stability and lead toxicity (*4*, *5*). To date, nearly all highly efficient PSCs have been produced using solution processing methods (*6*). Unfortunately, perovskite films obtained through this method with uncontrollable crystallization typically exhibit a high density of grain boundaries (GBs) and tend to accumulate various defects at GBs, resulting in severe nonradiative recombination of charge carriers. In general, GBs are unstable and susceptible to attack (*7*, *8*). In extreme environments, GBs can expedite the decomposition of perovskite by acting as reaction centers and channels for ion migration (*9*–*12*). Therefore, rational regulation to stabilize GBs is crucial for improving device stability.

In recent years, numerous strategies have been developed to optimize GBs to enhance the stability of perovskite films. One effective strategy involves the usage of additive engineering to select appropriate protective materials for safeguarding GBs. By using organic small molecules as additives, defects can be passivated, and a waterproof layer can be formed beneath the GBs, preventing water infiltration (*13*–*16*). Although these organic small molecules are primarily designed to enhance the humidity stability of PSCs, achieving operational stability for commercialized PSCs requires consideration of factors beyond humidity, such as thermal, electrical, and optical considerations (*17*, *18*). Polymers generally exhibit outstanding water-resistant, heat-resistant, and light-resistant properties (*19*, *20*). Hence, the polymerizable of organic additives represents a feasible approach to greatly improve the operational stability of PSCs under extreme conditions. In 2018, Li *et al*. (*21*) pioneered the incorporation of in situ polymerizable trimethallyl isocyanurate into the perovskite precursor solution. After the annealing process concludes, the crystallinity of the perovskite film improves, forming a cross-linked network structure at the GBs to stabilize the films and achieve PSCs with superior optoelectronic performance and operational stability. Furthermore, double[(3-methyloxetane-3-yl)methyl]thiophene-2,5-dicarboxylate can serve as an additive in perovskite precursor solution and polymerize at GBs during heat modification, forming a polymer structure. This polymer at GBs can release stress and passivate defects, thereby improving the device’s operational stability under adverse environmental conditions (*22*). However, these polymerizable small organic molecules often require higher temperatures or ultraviolet (UV) irradiation to initiate polymerization. In addition, most polymerizable organic additive monomers typically have a limited number of coordinating groups, reducing their ability to passivate various types of perovskite defects (*23*). Given these challenges, the development of a low-temperature, polymerizable, and multifunctional organic additive is critical for achieving efficient and operationally stable PSCs.

Although perovskite-based technologies generate less lead pollution compared to fossil fuel emissions, the potential long-term risks of lead leakage from degraded perovskite devices remain a critical concern for environmental and human safety. (*24*). To mitigate the risk of lead leakage caused by the degradation of PSCs, extensive research has been conducted to explore lead-free or partially lead-substituted perovskite materials, such as tin-based perovskites and double perovskite materials (*25*, *26*). However, tin-based perovskites exhibit poor stability, and the PCE of double PSCs remains relatively low (*27*, *28*). Therefore, despite the presence of certain drawbacks, lead-based PSCs continue to dominate due to their prevailing high efficiency and stability. In addition, ensuring high efficiency and stability is a prerequisite for eliminating lead; thus, minimizing lead release is of utmost importance. Recently, alternative methods have been developed to suppress the leakage of toxic lead in PSCs. For instance, polymer films (*4*), porphyrin derivatives (*29*), and others have been used for the external physical encapsulation of devices, demonstrating excellent ability to inhibit lead leakage. On the other hand, from a chemical perspective, anchoring lead and inhibiting the degradation of perovskite are also effective methods for preventing lead leakage (*30*). Perovskite films produced by the solution method inevitably result in the formation of uncoordinated Pb^2+^ at their GBs and surfaces. These deep-level defects contribute to an increase in open-circuit voltage (*V*_OC_) losses, consequently reducing the performance and stability of the devices (*31*, *32*). Therefore, chemically anchoring uncoordinated Pb^2+^ will reduce lead leakage and improve device performance (*33*, *34*). Now, small organic molecules are widely used to passivate uncoordinated Pb^2+^. However, the drawbacks of small organic molecules, such as high volatility, high diffusion coefficient, and uneven distribution in perovskites, often make it challenging to maintain high stability of PSCs under harsh operating conditions, further increasing the risk of lead leakage (*35*–*39*). Hence, there is an urgent need to optimize and stabilize GBs to obtain perovskite films with high quality, structural stability, and operational stability, with the aim of minimizing lead leakage in PSCs.

In this work, we developed a polymerization method to achieve highly stable and environmentally friendly high-performance PSCs. Through the addition of the small organic molecule *N*,*N*′-bis(acryloyl)cystamine (BAC) to the perovskite precursor solution, the terminal alkenes of BAC enable the construction of polymer BAC (PBAC) at the perovskite GBs during the annealing process of the perovskite films. PBAC not only overcomes the limitations of organic small molecules but also interacts with perovskites through its numerous functional groups and multiple reactive sites. This interaction effectively passivates deep-level defects, suppressing nonradiative carrier recombination. Furthermore, the PBAC-modified film demonstrated enhanced water resistance, effectively suppressing the migration of I^−^ and contributing to enhanced operational stability under extreme conditions. The PSCs with PBAC achieved an impressive PCE of 25.53% (0.1 cm^2^) (certified at 25.24%) and 24.03% at a larger area (1.0 cm^2^). Simultaneously, the unencapsulated devices maintained a value of more than 96% after continuous operation for 1500 hours under simulated AM 1.5 illumination. Furthermore, under damp heat conditions [85°C and 85% relative humidity (RH)], the encapsulated device retained more than 80% of its initial PCE during 2000 continuous hours of operation. Last, owing to the encapsulation of the polymer around the GBs and its anchoring ability to lead, the PBAC-modified PSCs exhibited a reduction in lead leakage. This research holds the promise of providing strategies for exploring high-performance and environmentally friendly PSCs.

## RESULTS

### Characterization of the PBAC polymer

**Fig. 1.**
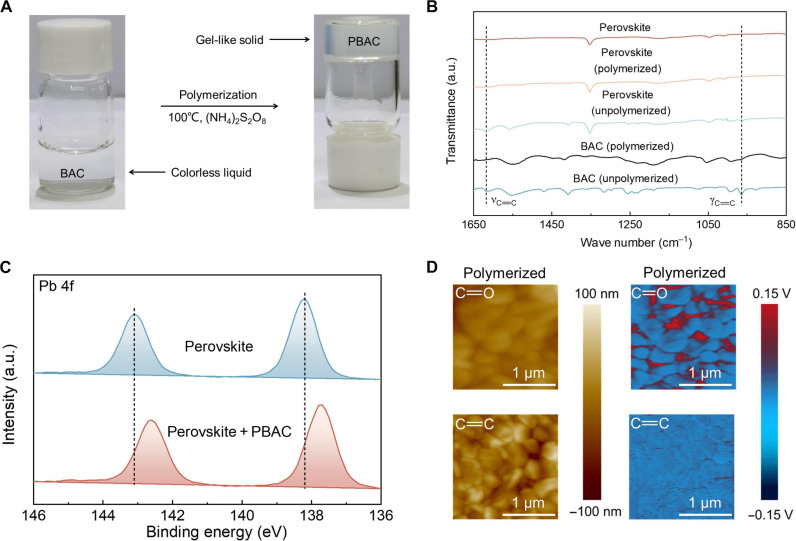
Molecular polymerization strategy. (**A**) Polymerization of BAC dissolved in *N*,*N*′-dimethylformamide (DMF)/dimethyl sulfoxide (DMSO) solvent under 100°C thermal treatment. (**B**) Fourier transform infrared (FTIR) spectra of perovskite films without and with PBAC modification. (**C**) XPS spectra of Pb 4f of the perovskite films without and with PBAC modification. (**D**) AFM morphology image (left) and corresponding infrared (IR) image (right) of the polymerized perovskite films at 963 and 1650 cm^−1^, corresponding to the C═C bond and C═O bond. a.u., arbitrary units.

To achieve the aforementioned functionalities, we introduced a thermally initiated polymerizable molecule containing amide groups, C═C bonds and C─S bonds, named BAC. As illustrated in Fig. 1A, at room temperature, BAC appears as a colorless liquid. Once BAC is heated at 100°C, it undergoes to form a gel-like solid. We anticipate that during the annealing process of perovskite films, BAC will form such a gel-like solid structure, providing additional chemical active sites, thereby passivating perovskite bulk defects and enhancing the performance and operational stability of the corresponding devices (*40*).

To investigate whether the gel-like solid is the result of BAC undergoing a polymerization reaction to form PBAC, we conducted gel permeation chromatography (GPC) measurements. As shown in fig. S1, when BAC is treated with ammonium persulfate as an initiator, its molecular weight increases, indicating that the gel-like solid formed by BAC upon heating and in the presence of an initiator is an oligomer. Furthermore, as exhibited in fig. S2, scanning electron microscopy (SEM) results reveal that the morphology of the gel-like solid formed by BAC molecules under heating and in the presence of an initiator undergoes a change compared to the individual BAC molecules, exhibiting a structure with pores. Therefore, in conjunction with the GPC measurement results, we anticipate that BAC can undergo a polymerization reaction to form PBAC under the conditions of an initiator and 100°C heating. On the basis of the measurement results from GPC and SEM, we inferred the reaction scheme and illustration for the BAC polymerization, as shown in fig. S3. The initiator (NH_4_)_2_S_2_O_8_ can decompose in the solvent to produce SO_4_^2−^ and NH_4_^+^. It decomposes under heating or in the presence of a catalyst to generate the free radical SO_4_•^−^. These radicals can attack the double bonds in the BAC molecules to form a new radical. Subsequently, this new radical can react with other BAC molecules to initiate chain growth. However, this growth is stochastic, and the exact structural composition cannot be fully determined. Therefore, on the basis of the experimental results, we have simply sketched a schematic diagram of the possible structures formed.

Subsequently, we used Fourier transform infrared (FTIR) spectroscopy and x-ray photoelectron spectroscopy (XPS) to elucidate the interplay between BAC molecules and perovskite. As shown in Fig. 1C, after introducing BAC into the perovskite precursor solution and annealing at 100°C, compared to the standalone BAC film, the stretching vibration peaks corresponding to the C═C bonds at 1615 and 964 cm^−1^ in the FTIR spectrum of the PBAC film disappear. Although this does not directly prove the occurrence of the polymerization reaction, in conjunction with the results from GPC and SEM, we believe that it can serve as a reference indicating the polymerization of BAC into PBAC. Simultaneously, fig. S4 (A to C) confirms the chemical interactions between BAC and perovskite. It can be observed that the C═O characteristic peak shifts from 1658 to a lower wave number of 1652 cm^−1^, the C─S characteristic peak shifts from 1190 and 809 cm^−1^ to 1185 and 804 cm^−1^, and the N─H characteristic peak shifts from 3248 to 3423 cm^−1^. This suggests that the chemical interactions between BAC and formamidium iodide (FAI) or BAC and lead(II) iodide (PbI_2_) involve hydrogen bonding between N─H and I or FA and interactions between C═O and Pb^2+^. In addition, in fig. S5, the survey XPS spectrum confirms that there is no transfer of foreign carbon, thus excluding the influence of charge on the results. Furthermore, after the introduction of BAC to form a polymer, the Pb 4f binding energy of the PBAC sample shifts toward a lower binding energy in comparison with that of the control sample (Fig. 1D), attributed to strengthened chemical interactions between PBAC and perovskite. In addition, the two S 2p peaks in PBAC, located at 164.8 and 163.6 eV (fig. S6A), shift to 164.5 and 163.3 eV, respectively, indicating chemical interaction between the sulfur atoms and lead ions (*41*, *42*). The shift in the I 3d peak (fig. S6B) is induced by hydrogen bonding between PBAC and the [PbI_6_]^4−^ octahedra. After polymerization of BAC within the perovskite film, the shift in the N 1s peak at 399.5 eV further confirmed the existence of N─H···I hydrogen bonds (fig. S6C). Then, we used liquid-state proton nuclear magnetic resonance (NMR) spectroscopy to investigate chemical interactions in the precursor solution. The results presented in fig. S7A demonstrate that the introduction of PbI_2_ into the BAC solution leads to smaller chemical shifts in the corresponding ^13^C peaks compared to the original BAC. This observation suggested the occurrence of chemical interactions between BAC and PbI_2_. Subsequently, when FAI was added into the BAC solution, a lower ^1^H peak chemical shift was observed, further demonstrating the hydrogen bonding interactions between BAC and FAI (fig. S7B).

Further insights into the distribution and locations of BAC in perovskite films were gained through atomic force microscopy–based infrared (AFM-IR) spectroscopy (*43*). Figure S8 illustrates the AFM morphology image (left) and corresponding infrared (IR) image (right) of the C═C and C═O characteristic peaks at 963 and 1650 cm^−1^ in control and BAC-modified perovskite films without annealing. The red region signifies BAC molecules at the corresponding IR characteristic peak positions, while the deep blue region corresponds to the perovskite. As shown in fig. S8A, the control perovskite films only exhibited the presence of deep blue region, which showed no response to C═C and C═O groups. In contrast, in fig. S8B, we observed the presence of the red region at the GBs adjacent to the perovskite, indicating the simultaneous existence of C═C and C═O groups. This also suggests that after the addition of BAC to form the perovskite films, it does not polymerize initially but rather exists in molecular form at the perovskite GBs. In Fig. 1E, for the perovskite films formed with the addition of BAC molecules and subsequent annealing treatment, only the presence of C═O groups at the GBs was observed, with a clear absence of C═C groups. This indicates that after prolonged heating and annealing, BAC molecules can polymerize to PBAC at the GBs of the perovskite film. In addition, upon the introduction of the PBAC, the conductivity of the perovskite films almost remains unchanged (fig. S9). This further indicates that the formed polymer does not hinder the transport of charge carriers in the perovskite.

Furthermore, to determine the impact of PBAC on the crystallization of perovskite films, we conducted SEM characterization (fig. S10, A and B). In comparison with that of the control films, the grain size of the PBAC-modified perovskite film experiences a slight growth. Meanwhile, the increased intensity of the characteristic peaks in the x-ray diffraction (XRD) patterns corresponding to the perovskite films (fig. S11A) further suggested an enhancement in the crystallinity of the PBAC perovskite films. Simultaneously, there is no change in the positions of the corresponding characteristic peaks (fig. S11B), indicating that PBAC does not infiltrate the perovskite lattice, as evidenced by the consistent bandgap of the perovskite films before and after modification with PBAC in the *T*_auc_ plot results (fig. S12). All the above characterizations confirm that the presence of PBAC is conducive to enhancing the crystallinity of the perovskite films.

### Photovoltaic performance and optoelectronic properties of PSCs

**Fig. 2.**
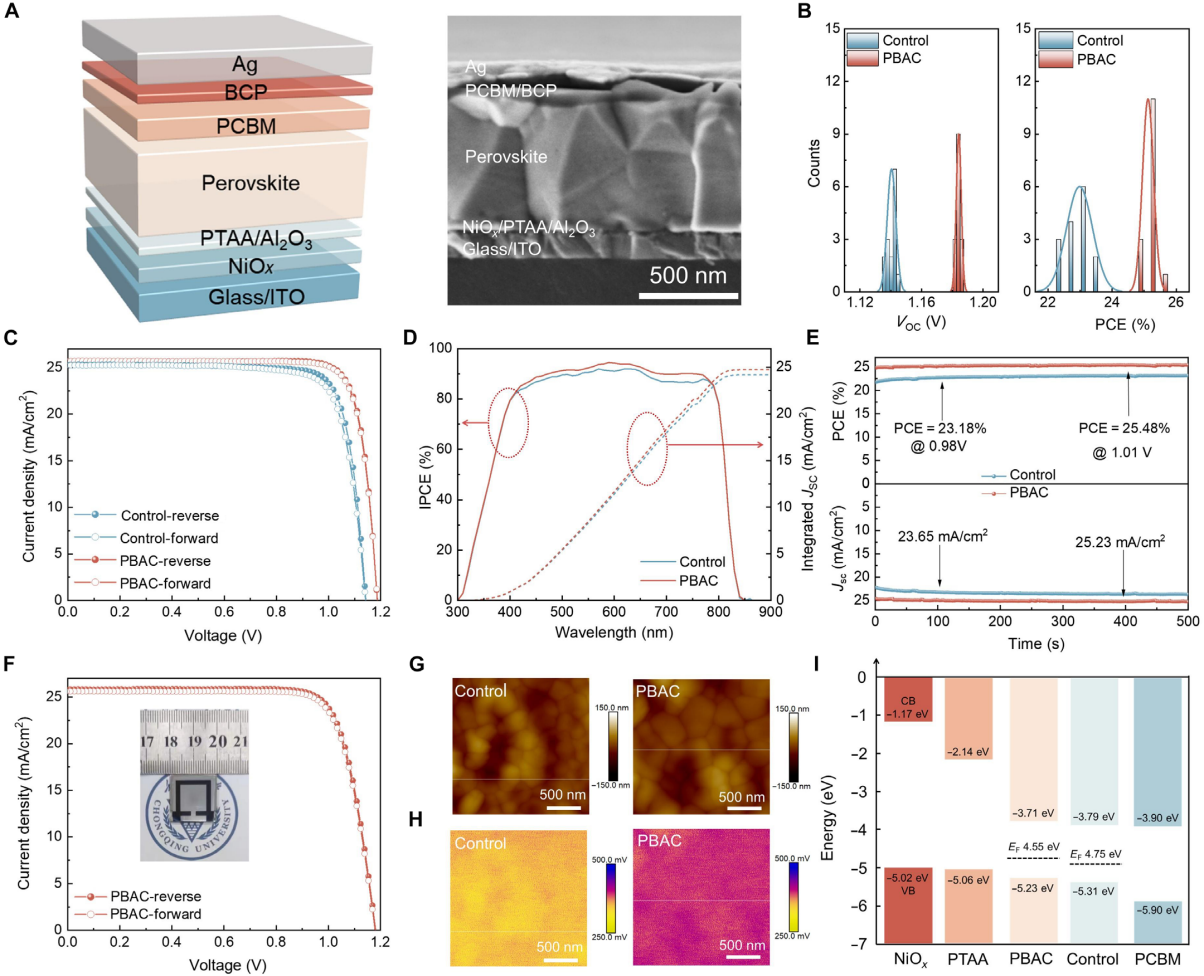
Device performance and optoelectronic properties. (**A**) Device structure diagram of the PSCs and corresponding cross-sectional SEM image. (**B**) PCE and *V*_OC_ histogram of the control and PBAC-modified devices. (**C**) Photovoltaic performance characterization. *J*-*V* curves of the optimized devices without and with PBAC modification. (**D**) IPCE curves and integrated *J*_SC_ of the PSCs without and with PBAC modification. (**E**) *J*_SC_ and PCEs at the MPP for the control and PBAC-modified devices. (**F**) *J*-*V* curve of the optimized PBAC-modified devices with an active area of 1.0 cm^2^. (**G**) AFM image of perovskite films without and with PBAC modification. (**H**) KPFM image of perovskite films without and with PBAC modification. (**I**) Energy level diagram.

To comprehensively investigate the impact of PBAC on the performance of PSCs, we designed a structurally straightforward planar device comprising indium tin oxide (ITO)/NiO*_x_*/poly(triaryl amine) (PTAA)/Al_2_O_3_/perovskite/phenyl-C61-butyric acid methyl ester (PC_61_BM)/bathocuproine (BCP)/Ag (Fig. 2A). By optimizing the introduction of BAC into the perovskite precursor solution, the optimal concentration was determined to be 0.03 mM (fig. S13). Subsequently, analyzing the PCE distribution of 15 individual devices, the average PCE increased from 22.98% (control) to 25.12% (PBAC), providing further confirmation of the advantages and reproducibility of the PBAC modification in the preparation of high-performance PSCs (Fig. 2B and fig. S14). Notably, a small amount of (NH_4_)_2_S_2_O_8_ is used to facilitate polymerization without affecting film crystallization and device performance (figs. S15 and S16). Figure 2C illustrates the optimized *J-V* curves of the control and PBAC devices under standard AM 1.5G illumination. The control devices displayed a PCE of 23.44%, a *V*_OC_ of 1.143 V, a short-circuit current density (*J*_SC_) of 25.31 mA/cm^2^, and a fill factor (FF) of 81.0%. The optimized PBAC-modified devices achieved a maximum PCE of 25.53%, *V*_OC_ of 1.186 V, *J*_SC_ of 25.68 mA/cm^2^, and FF of 83.8% (table S1). The best-performing device underwent certification at the Test and Calibration Center of New Energy Device and Module, Shanghai Institute of Microsystem and Information Technology, Chinese Academy of Sciences, resulting in a PCE of 25.24% (*J*_SC_ = 25.60 mA/cm^2^, *V*_OC_ = 1.179 V, FF = 83.68%) (fig. S17). The notable improvement of the PCE is mainly ascribed to enhancements in *V*_OC_ and FF, stemming from the suppression of nonradiative carrier recombination and enhancement in carrier transport at the GBs (*44*). In addition, the incident photon-to-electron conversion efficiency (IPCE) spectra of the PSCs are depicted in Fig. 2D, showing that the calculated integrated photocurrent densities for the control and PBAC-modified devices are 24.17 and 24.73 mA/cm^2^, respectively, which are consistent with the *J*_SC_ obtained from the *J-V* curves. Subsequently, maximum power point (MPP) tracking was carried out to accurately evaluate the true output efficiency of the PSCs. As shown in Fig. 2E, the PBAC devices stably outputted at 25.48% (*V*_MPP_ = 1.01 V) for 500 s; in contrast, the control devices exhibited an output efficiency of only 23.18% (*V*_MPP_ = 0.98 V). Last, as presented in Fig. 2F and table S2, the PBAC-modified devices achieve an optimized PCE of 24.03% (a *V*_OC_ of 1.181 V, a *J*_SC_ of 25.81 mA/cm^2^, and an FF of 78.8%) at a larger area (with an aperture area of 1.0 cm^2^) with negligible hysteresis. Upon scaling up the aperture area of the device to 1.0 cm^2^, the FF of the device is reduced because of the increase in series resistance. Therefore, we have redesigned two rectangular structures to investigate the impact of different configurations on PSC performance. As seen in fig. S18A and table S3, the device based on this rectangular structure exhibits a PCE of 22.92%, with a *V*_OC_ of 1.178 V, a *J*_SC_ of 25.68 mA/cm^2^, and an FF of 75.7%. Furthermore, the device with the rectangular structure shown in fig. S18B and table S4 exhibits a PCE of 23.56%, a *V*_OC_ of 1.180 V, a *J*_SC_ of 25.72 mA/cm^2^, and an FF of 77.6%. The PCE and FF of both rectangular structures are lower than those of the original design (Fig. 2F), indicating the necessity for a more meticulously designed rectangular configuration to enhance the FF and PCE of PSCs.

Stable photoluminescence (PL) and time-resolved photoluminescence (TRPL) spectroscopy were used to explore the carrier dynamics in the perovskite films. As shown in fig. S19A, when the perovskite film is coated onto pure glass, the PL intensity of the PBAC-modified perovskite film is markedly greater than that of the control sample, indicating that the introduction of PBAC promotes an improvement in the film quality. The conclusions are further confirmed by the TRPL results (fig. S19B). For the control perovskite film, the average carrier lifetime (τ_ave_) is calculated to be 308.35 ns. However, for the PBAC-modified perovskite film, this value increases to 1046.44 ns (table S5). The carrier lifetime exceeding the microsecond range suggests that PBAC enhances the quality of perovskite films, simultaneously passivating defects and suppressing nonradiative recombination, leading to improved *V*_OC_ (*45*, *46*).

The photovoltaic characteristics of PBAC-modified devices were systematically explored to uncover the mechanisms contributing to enhanced photovoltaic performance. The ideal factor (*m*) was obtained by fitting the data points of *V*_OC_ versus light intensity for the control and PBAC-modified devices (fig. S20). The ideality factor of the PBAC-modified devices is 1.13, which is lower than the value of 1.60 for the control device, indicating reduced carrier nonradiative recombination losses and, consequently, lower *V*_OC_ losses in the device. The built-in potential (*V*_bi_) of PSCs can be determined from the Mott-Schottky (M-S) plot, as depicted in fig. S21. It can be discerned that the *V*_bi_ of the control devices is 1.08 V while that of the PBAC devices increases to 1.17 V, suggesting an enhanced driving force for the separation of photogenerated carriers in accordance with the ongoing amelioration of *V*_OC_ (*47*). In conclusion, the optimized photovoltaic performance of the PBAC-modified devices primarily arises from the improvements in carrier extraction efficiency, prolonged carrier lifetime, reduced defect density, and inhibited nonradiative recombination, resulting in elevated *V*_OC_ and FF.

The surface potential of the perovskite films was measured using Kelvin probe force microscopy (KPFM). Initially, on the same test structure, direct contact with the perovskite surface can be achieved through an AFM probe, as illustrated in Fig. 2G. Subsequently, the corresponding surface potential differences of the perovskite films are obtained. As shown in Fig. 2H, the surface potential of the PBAC-modified films increased relative to that of the control films. According to the relevant literature, this is attributed to the observable chemical interaction induced by the C═O and C─S groups in PBAC with uncoordinated Pb^2+^ (*48*). In addition, UV photoelectron spectroscopy (UPS) was used to examine alterations in the energy levels of the devices. As shown in fig. S22, the complete UPS spectrum shows that there is no new characteristic peak. Furthermore, under PBAC modification, the valence band energy (*E*_VB_) of the perovskite films shifts from −5.31 eV in the control films to −5.23 eV in the PBAC films (fig. S23 and table S6). Figure 2I reveals that after PBAC was introduced, there was an increase in the clarity of the alignment of energy levels between the perovskite and the transport layer, contributing to improved charge carrier extraction and transport, which aligns well with the M-S analysis. Moreover, related studies suggest that residual stress can decrease device stability and PCE (*49*). The improvement in crystallization of PBAC films and the decrease in defects could be the reasons for the alleviation of residual stress. The reduced residual stress will contribute to enhancing the device’s PCE and stability (*50*, *51*).

### Harsh environment stability and long-term operation stability of PSCs

**Fig. 3.**
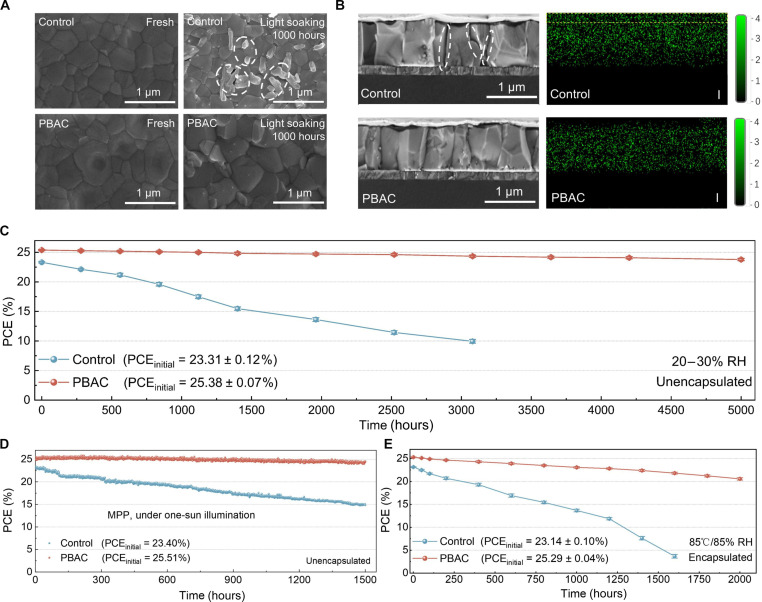
Stability. (**A**) Top-view SEM images of the control and PBAC-modified perovskite films under fresh conditions and after aging under light soaking conditions. (**B**) Cross-sectional images and corresponding energy-dispersive spectroscopy mapping images of the control and PBAC-modified perovskite films modified after aging for 1000 hours under continuous of 1-sun illumination. (**C**) Humidity stability of the unencapsulated control and PBAC-modified devices aged at room temperature under 20 to 30% RH. (**D**) Stability evolution of the unencapsulated control and PBAC-modified devices measured at MPP under continuous 1-sun illumination in N_2_. (**E**) Exposure of the encapsulated control and PBAC-modified devices to 85°C and 85% RH under dark conditions. The error bar is the SD of the three devices. Data are presented as means ± SEM.

Apart from evaluating the device performance, ensuring the stability of PSCs is equally imperative. Therefore, we assessed the stability of perovskite films in harsh environments. The characterization of water contact angle can evaluate the water permeability and moisture resistance of perovskite films (fig. S24). Compared with the 53.1° of the control film, the water contact angle of the film modified by PBAC increased to 80.3°, which indicated the excellent hydrophobicity of PBAC. First, the microscopic degradation of the perovskite films was characterized by SEM after aging for 2000 hours at an RH of 50 to 60%. The degradation of the control films is pronounced, with the generation of an amount of PbI_2_, which is undoubtedly fatal to device stability (fig. S25A). Encouragingly, the PBAC-modified films showed minimal changes (fig. S25B), attributed to the hydrophobic PBAC at the GBs and increased of crystallinity and film densification induced by PBAC modification. In addition, the XRD patterns after aging under the same conditions reveal a decrease in the intensity of the PbI_2_ characteristic peak at 2θ = 12.73° for the PBAC perovskite films compared to that of the control samples (fig. S26A). Meanwhile, the UV-visible (UV-vis) absorption spectra of the perovskite film after aging (fig. S27A) showed a smaller change in absorbance for the PBAC-modified film over time. This indicates that the hydrophobic PBAC formed at the GBs, along with the decrease in the number of GBs and the improvement in film quality, is the reason for the stability of the PBAC sample under high humidity. Subsequently, the photostability and thermal stability of the perovskite films were also assessed. After aging for 1000 hours at 60°C in a nitrogen atmosphere, negligible sporadic PbI_2_ formation is observed for the PBAC-modified films, whereas the morphology of the control perovskite films exhibits changes and nearly complete degradation (fig. S28, A and B). According to the XRD patterns, the control films exhibit the presence of PbI_2_ and δ-phase perovskite (fig. S26B). In addition, the intensity of the absorbance for the control film is much lower than that of the PBAC film (fig. S27B). Photostability was assessed by aging the perovskite films under solar irradiation for 1000 hours in a nitrogen atmosphere. The surface morphology of the PBAC-modified films shows almost no change, while the control perovskite films clearly produce PbI_2_ (Fig. 3A). The XRD (fig. S26C) and UV-vis absorption results (fig. S27C) were consistent with the SEM results. The improved stability of the PBAC sample under light and heat is closely related to the reduction in I vacancies (*V*_I_) within its perovskite film (*52*). Under typical light and heat conditions, the formation of *V*_I_ defects is easily triggered, amplifying ion migration within the perovskite and initiating chemical chain reactions, resulting in perovskite degradation. This property poses a threat to the long-term stability of PSCs (*53*). As shown in Fig. 3B, after continuous solar irradiation for 1000 hours in a N_2_ atmosphere, substantial diffusion of I^−^ into the Ag electrode region occurs in the control devices, while the PBAC-modified devices exhibited much weaker diffusion, further indicating the inhibition of ion migration due to the interaction between PBAC and perovskite. In addition, evident perovskite degradation fractures are observed in the control films after aging (dashed outline). In contrast, the PBAC-modified perovskite films remain essentially continuous, providing further evidence that the PBAC-modified strategy contributes to the improved stability of perovskite films.

Furthermore, as depicted in Fig. 3C, the storage stability of the unencapsulated PSCs was evaluated under a relatively dry air environment (RH, 20 to 30%). After 5000 hours of aging, the PBAC-modified device maintains more than 94% of its initial value, while the control device exhibits a decline in its PCE to the initial 43% after aging for 3000 hours. This favorable outcome has bolstered our confidence in evaluating the operational stability of PSCs under extreme conditions. Under constant simulated AM 1.5 illumination (100 mW/cm^2^) conditions, we evaluated the operational stability of both the unpackaged control device and the PBAC-modified device by MPP tracking. As shown in Fig. 3D, after continuous testing for 1500 hours, the PBAC-modified device sustains more than 96% of its initial PCE at 25.50%, in contrast to the control device, which degrades to 63% of its initial PCE at 23.40%. In addition, following damp heat aging for the encapsulated devices under 85°C and 85% RH conditions, the control device markedly drops to 15% of its initial PCE after 1600 hours of aging; in contrast, the PBAC-modified device preserves more than 80% of its initial PCE after 2000 hours of aging (Fig. 3E). In conclusion, GBs are prone to attack owing to the presence of defects. Under extreme conditions involving humidity, light, and heat, GBs create channels for water molecule penetration and ion migration, leading to accelerated degradation of perovskite (*40*, *54*, *55*).

### Pb leakage analysis of PSCs

**Fig. 4.**
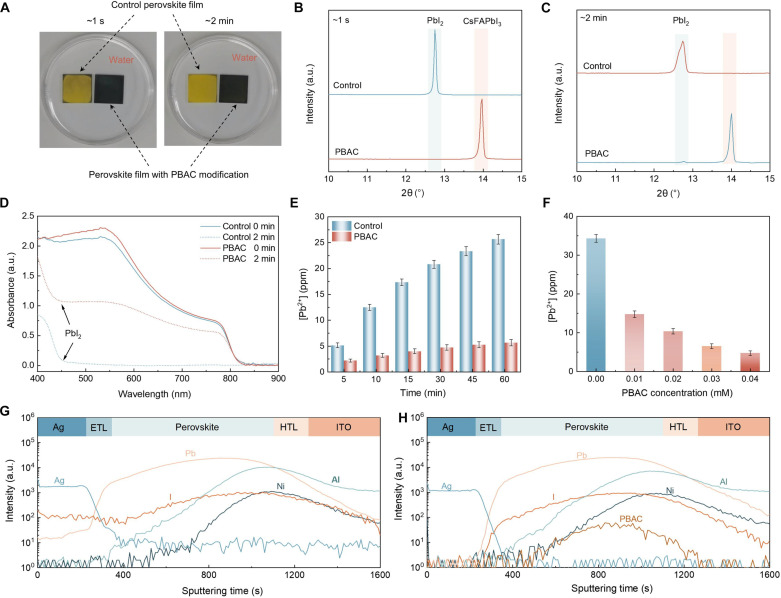
Pb leakage analysis. (**A**) Photographs of water immersion measurements on control and PBAC-modified perovskite films. (**B** and **C**) XRD patterns of aged perovskite films after (B) ~1 s and (C) ~2 min of water soaking. (**D**) UV-vis spectra of the control and PBAC-modified perovskite films before and after aging under deionized water immersion. (**E**) Variation in the Pb^2+^ concentration under room temperature conditions during immersion in deionized water. (**F**) Leaked lead concentration after immersing the PSCs with a different PBAC dosage into acidic water for 480 min. (**G** and **H**) TOF-SIMS analysis of the PSCs (G) without and (H) with PBAC modification after aging for 1000 hours at 50 to 60% RH. ETL, electron transport layer; HTL, hole transport layer.

Because of the robust coordination capability of PBAC with Pb^2+^ and the high humidity stability of PBAC perovskite films, we anticipate the potential of this material to attenuate Pb leakage in devices. As shown in Fig. 4A, we first observed the experimental image of perovskite films immersed in water, and it can be seen that the control films rapidly decompose into PbI_2_ upon immersion in water, which becomes more evident after 2 min of immersion. Conversely, because of the hydrophobic PBAC encapsulating the GBs, PBAC perovskite films can maintain the black perovskite phase even after immersion in water. In addition, the XRD patterns of perovskite films after different durations of immersion in water (Fig. 4, B and C) further confirm the effective protection of GBs by the polymer formed by PBAC. Subsequently, we characterized the perovskite films immersed in water using UV-vis spectroscopy, as shown in Fig. 4D. The results indicate a decrease in the absorption intensity of the reference perovskite film after 2 min of water immersion, accompanied by the generation of PbI_2_, while the PBAC perovskite film maintains the perovskite phase even after prolonged immersion without decomposing into PbI_2_. These findings suggest that the encapsulation of GBs by PBAC can effectively hinder direct contact and interaction between water and perovskite, preventing the collapse of the perovskite structure and thereby decelerating the escape of Pb^2+^ (*56*). To further quantify the Pb^2+^ leaching rate, we conducted inductively coupled plasma optical emission spectrometry (ICP-OES) on a 5-ml water solution containing devices, as illustrated in Fig. 4E. After immersing the control devices at room temperature for 480 min, the Pb^2+^ concentration is 127.83 parts per million (ppm) (average), while the PBAC devices under the same conditions exhibit a Pb^2+^ concentration of 35.90 ppm (average), reducing nearly 72% lead leakage in PSCs, confirming the effective reduction in lead leakage by the polymer formed by PBAC. In addition, with an increase in PBAC concentration, we observed a decreasing trend in Pb^2+^ concentration (Fig. 4F). Because of the higher concentrations, PBAC-modified perovskite films formed with increased hydrophobicity. We performed time-of-flight secondary ion mass spectrometry (TOF-SIMS) to investigate lead leakage in PSCs. After aging for 1000 hours at 50 to 60% RH, the control devices display a prominent lead signal at the PCBM and the Ag electrode (Fig. 4G), indicating that the perovskite is vulnerable to degradation into PbI_2_ and accelerates lead leakage under high humidity (*57*). In contrast, the Pb signal intensity in the PBAC devices is low (Fig. 4H), confirming the trend of inhibited ion diffusion due to the stabilization of GBs by the PBAC under high humidity. In summary, the construction of a PBAC polymer at perovskite GBs not only prevents water erosion but also substantially mitigates lead leakage in devices.

## DISCUSSION

In conclusion, we successfully developed a method for synthesizing the polymer PBAC by a low-temperature thermally induced strategy. PBAC, which features numerous hydrogen bonds and functional groups such as C═O and C─S, establishes strong interactions with perovskite, yielding high-quality perovskite films with improved crystallinity, lower defect density, and inhibited ion migration. This results in a substantial enhancement in the PCE and operational stability. The champion PCE of PBAC-based PSCs reaches 25.53% (0.1 cm^2^) and 24.03% (1.0 cm^2^). Furthermore, the modified PSCs displayed a remarkable improvement in operational stability under MPP tracking, maintaining 96% of the initial value after 1500 hours. In addition, after aging for 2000 hours at 85°C and 85% RH, more than 80% of the initial PCE was retained. The polymer hydrophobicity prevents Pb^2+^ from dissolving in water, effectively reducing lead leakage. This work provides an effective approach to simultaneously prevent lead leakage and enhance the long-term operational stability of devices, thereby facilitating the widespread deployment of efficient PSCs in clean energy applications.

## MATERIALS AND METHODS

### Materials

*N*,*N*′-dimethylformamide (DMF; 99.8%), dimethyl sulfoxide (DMSO; 99.8%), chlorobenzene (CB; >99.9%), isopropanol (IPA; 99.5%), and Al_2_O_3_-dispersed solution (20 wt % in IPA) were purchased from Sigma-Aldrich. FAI (99.9%), cesium iodide (CsI; 99.999%), and PbI_2_ (99.999%) were purchased from Advanced Election Technology Co. Ltd. PTAA (molecular weight distribution: 6000 to 15,000) and BCP were purchased from Xi’an Polymer Light Technology Corp. BAC (99.0%) and ammonium persulfate [(NH_4_)_2_S_2_O_8_, >99.0%] were purchased from Macklin Biochemical Technology Co. Ltd. Nickel oxide (NiO*_x_*) was synthesized according to the previous work (*58*). The chemicals and solvents were used without undergoing additional purification procedures.

### Device fabrication

The ITO glass underwent cleaning in an ultrasonic bath sequentially with detergent, deionized water, acetone, and IPA. The ITO substrates were then subjected to UV ozone exposure for 30 min. The NiO*_x_* powder was dispersed in deionized water, resulting in a concentration of 25 mg/ml after preparation and then followed by spin coating on the ITO substrates at 5000 rpm for 30 s. The NiO*_x_* films underwent annealing on a heating plate at 150°C for 10 min in ambient air, followed by immediate transfer into a glove box. After that, a PTAA solution (2 mg in 1 ml of CB) was spin-coated onto NiO*_x_* films at 6000 rpm for 30 s. Subsequently, following a 5-min ultrasonication of the Al_2_O_3_ dispersion solution (0.4 wt % in IPA), it was spin-coated onto the PTAA films at 5000 rpm for 30 s. To prepare the precursor solution of FA_0.95_Cs_0.05_PbI_3_ perovskite, 228.7 mg of FAI, 18.2 mg of CsI, and 645.4 mg of PbI_2_ were dissolved in a mixed solvent of DMF/DMSO (with a volume ratio of 4:1). The BAC molecule was added to the above solution (0 to 0.04 mM), and a small amount of (NH_4_)_2_S_2_O_8_, corresponding to 1 mol % of BAC, was simultaneously introduced to facilitate the polymerization process. Afterward, spin coating the perovskite precursor solution onto the glass/ITO/NiO*_x_*/PTAA/Al_2_O_3_ substrate occurred at 2000 rpm for 10 s, followed by a subsequent spin coating at 4000 rpm for 40 s. In the second spin-coating step, 150 μl of CB was dropped onto the perovskite film 5 s before the end of the program. The resultant wet perovskite films were subjected to annealing at 100°C for a duration of 30 min. Following that, a solution with a concentration of 23 mg/ml PC_61_BM in CB was applied to the perovskite films through spin coating at 2500 rpm for 40 s. Following that step, a solution consisting of BCP (0.5 mg/ml) in IPA was spin-coated onto the PC_61_BM films at 5000 rpm for 30 s. The last step involved the thermal evaporation of a 100-nm silver electrode under a vacuum of 5 × 10^−5^ Pa.

### Characterization of perovskite films

The surface morphology and cross section of the perovskite films were investigated through characterization using SEM (Apreo S HiVac FEI). The XRD patterns were obtained using a Rigaku Ultima IV, which was equipped with Cu Kα radiation (λ = 1.5406). Using a Shimadzu UV3600 spectrophotometer, measurements were performed to obtain the UV-vis absorption spectra of the perovskite films. Using the Nicolet iS50 FTIR spectrometer, FTIR spectra were recorded. The Thermo Fisher Scientific ESCALAB 250Xi spectrometer was used for XPS measurements, using a monochromatic Al Kα source with an energy of 1486.6 eV. The steady-state PL spectra were acquired using a fluorescence spectrometer (FLS1000, Edinburgh Instruments Limited) with a 480-nm excitation light source. TRPL spectra were recorded on a fluorescence spectrophotometer (FLS1000, Edinburgh Instruments Limited) under the excitation of a pulsed laser with a wavelength of 450 nm.

### Characterization of the solar cells

The *J-V* characteristics of devices were evaluated under ambient air conditions (with an RH of 30 to 50%) using a Newport-2612A solar simulator and a Keithley 2400 source meter. By using a black metal mask, the effective area of the cell was established as either 0.1 or 1.0 cm^2^. The measurement of IPCE was conducted using the Newport instrument system (Newport-74125), which was equipped with a lock-in amplifier and a 300-W xenon lamp.

### Characterization of the device stability

The operational stability measurement of the unencapsulated devices was conducted under a continuous 1-sun illumination (a light-emitting diode lamp without UV filter) at 55° ± 5°C at MPP using an MPP trace system (YH-VMPP-16) in a N_2_-filled glove box for 1500 hours. The evaluation of damp heat stability for encapsulated devices was carried out in a constant temperature and a humidity chamber (ZK-301) under conditions of 85°C and 85% RH.

### ^1^H and ^13^C NMR spectra measurements

Using the Bruker Advanced III 400 MHz spectrometer, ^1^H and ^13^C NMR spectra were obtained. The samples were dissolved in deuterated DMSO.

### AFM-IR measurements

AFM-IR experiments were conducted using the NanoIR3-FS AFM test system, integrating nano-FTIR with AFM. It features an AFM microscope in contact mode and a quantum cascade laser (LS-HS-QCL). The IR measurement wave number is tunable within the range of 800 to 1800 cm^−1^.

### M-S measurements

Under conditions of darkness, the M-S measurement was executed using the Chenhua electrochemical workstation (CHI 760E).

### KPFM measurements

KPFM measurements were carried out by sensing and counteracting the Coulombic forces between the probe (nanosensor, polymer-based piezoresistive probe-electrostatic force microscopy, chromium/gold coating) and the sample. This allowed for the determination of the contact potential difference between the probe and the sample.

### TOF-SIMS measurements

The PHI nanoTOF II instrument was used for TOF-SIMS measurements, using pulsed monoatomic ions from an oxygen ion beam (1 keV) for sputtering and a pulsed Bi_3_^++^ ion beam (30 keV) for analysis.

### ICP-OES measurements

ICP-OES measurements were carried out on an Agilent 5110 (OES) to measure the Pb^2+^ concentration in water.
