# Supplementary material for: Molecular polymerization strategy for stable perovskite solar cells with low lead leakage
Source: Sci Adv. 2025 May 7;11(19):eado7318. doi: 10.1126/sciadv.ado7318 (PMC12057662; doi:10.1126/sciadv.ado7318)
Supplement: Supplementary file 1 — Figs. S1 to S28 Tables S1 to S6 [file sciadv.ado7318_sm.pdf]

Science Advances
AAAS

Supplementary Materials for

Molecular polymerization strategy for stable perovskite solar cells with low lead leakage

Qixin Zhuang *et al.*

Corresponding author: Xiong Li, xiongli@hust.edu.cn; Zhigang Zang, zangzg@cqu.edu.cn

Sci. Adv. **11**, eado7318 (2025)
DOI: 10.1126/sciadv.ado7318

This PDF file includes:

Figs. S1 to S28
Tables S1 to S6

1. Supplementary figures

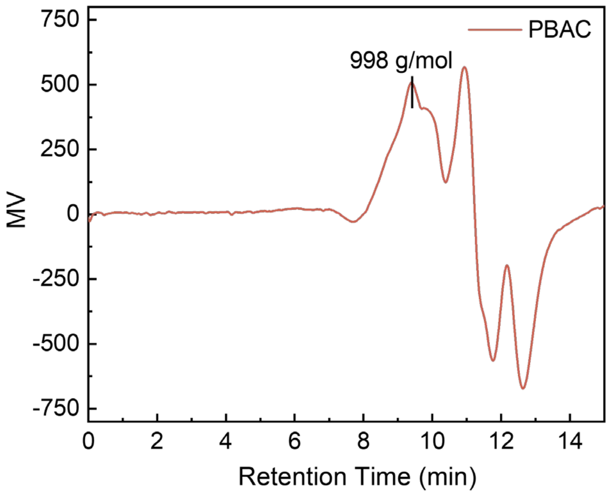

Fig. S1. GPC measurement of PBAC. GPC was measured using chloroform as the mobile phase.

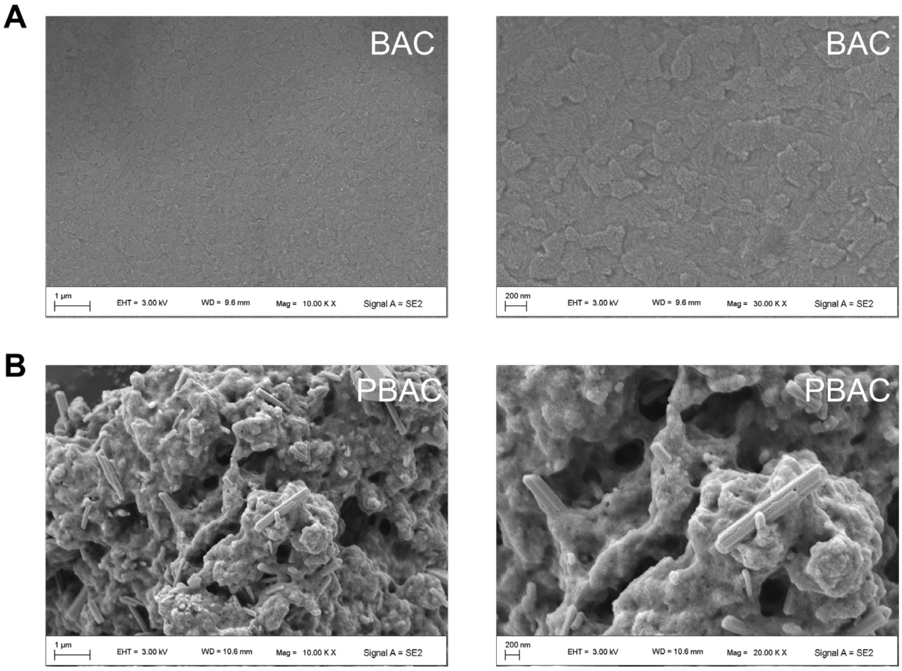

Fig. S2. Morphology of films. SEM images of (**A**) BAC and (**B**) BAC polymerization to PBAC films.

Firstly, APS decomposes under heating conditions to generate free radicals:

$$(NH_4)_2S_2O_8 \longrightarrow 2NH_4^{+} + 2SO_4^{2-} \qquad SO_4^{2-} \xrightarrow{100°C} SO4•^{-} + e^{-}$$

Here SO4•⁻ stands for sulfate radical. Sulfate radical can attack the double bond in BAC molecule and form a new radical:

$$SO4•^{-} + CH_2{=}CH{-}C{=}O{-}NH{-}CH_2{-}CH_2{-}NH{-}C{=}O{-}CH{=}CH_2 \longrightarrow CH_2\cdot{-}CH{-}C{=}O{-}NH{-}CH_2{-}CH_2{-}NH{-}C{=}O{-}CH{=}CH_2$$

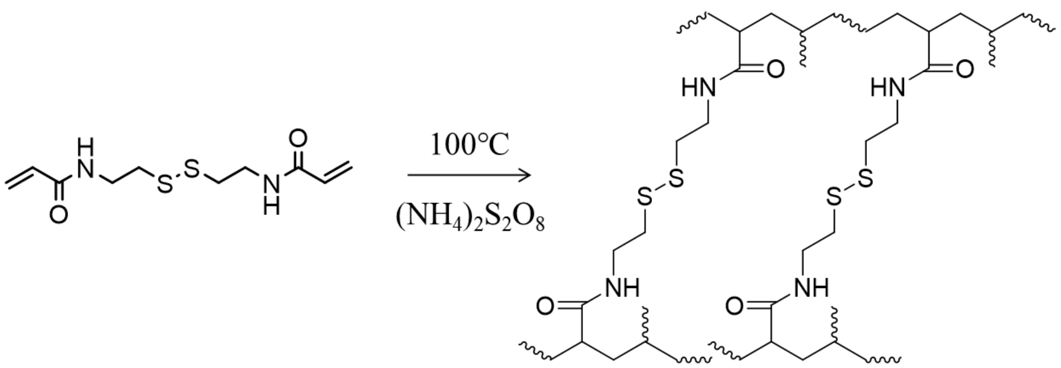

Fig.S3. Schematic diagram of BAC polymerization reaction.

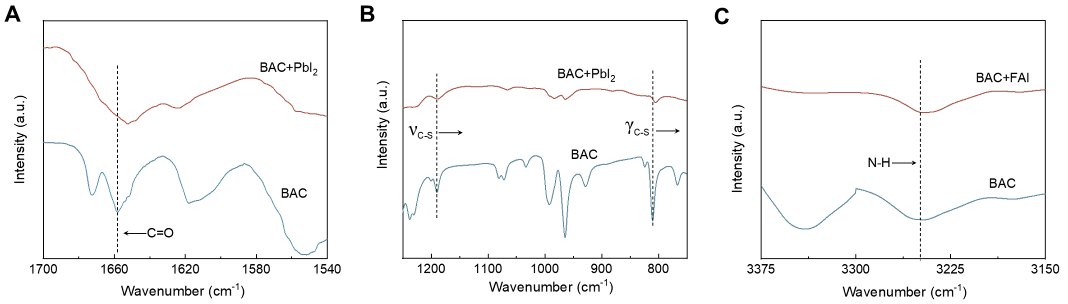

Fig. S4. Fourier-transform infrared spectroscopy spectra of thin films. (**A**) and **(B)** BAC and BAC+PbI_2 films, (**C**) BAC and BAC+FAI films.

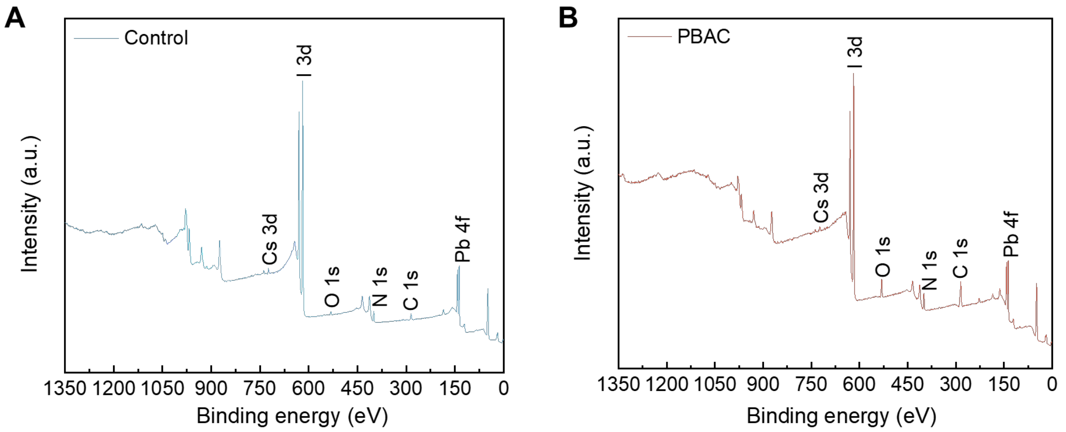

Fig. S5. XPS spectra of perovskite films. (**A**) control and (**B**) PBAC modified perovskite films.

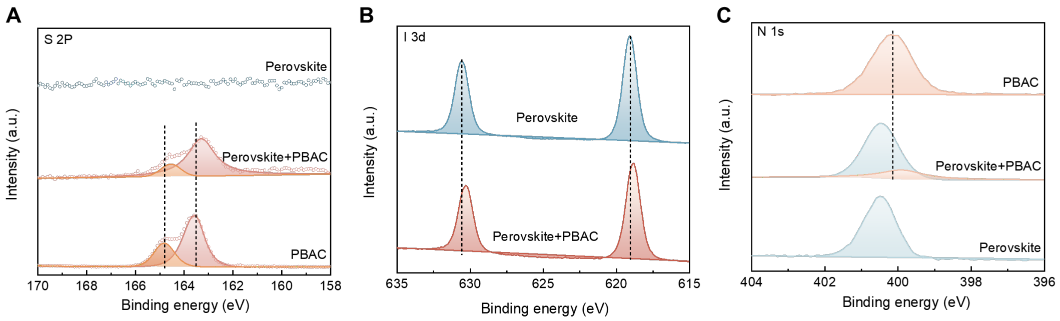

Fig. S6. XPS spectra of perovskite films. (**A**) S 2p, (**B**) I 3d and (**C**) N 1s of the perovskite films without and with PBAC modification.

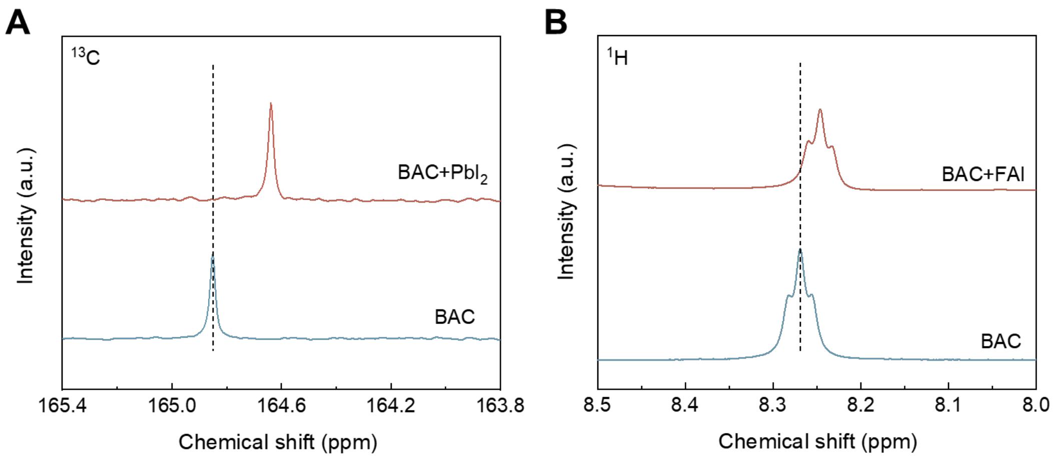

Fig. S7. NMR spectra obtained from the reaction of BAC, PbI_2 and FAI in DMSO-d_6. (**A**) ^{13}C NMR spectrum (BAC and BAC+PbI_2). (**B**) ^{1}H NMR spectrum (BAC and BAC+FAI).

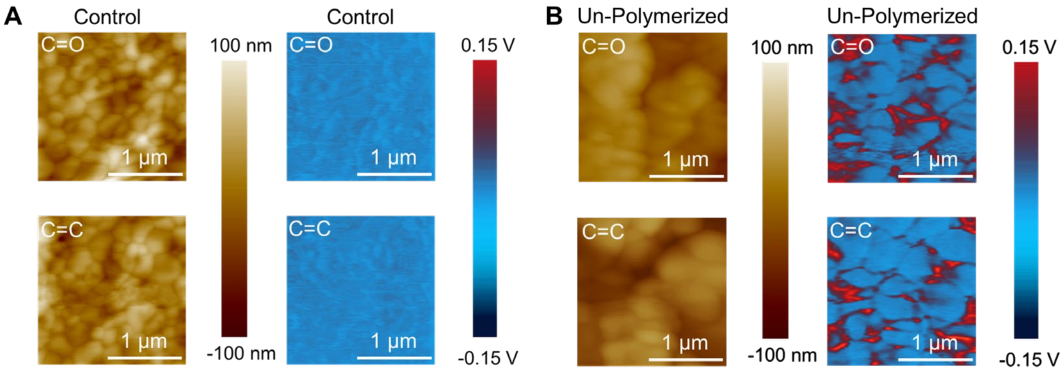

Fig. S8. AFM-IR analysis. AFM morphology image (left) and corresponding IR image (right) of the **(A)** control and **(B)** un-polymerized perovskite films at 963 cm^{-1} and 1650 cm^{-1}, corresponding to the C=C bond and C=O bond.

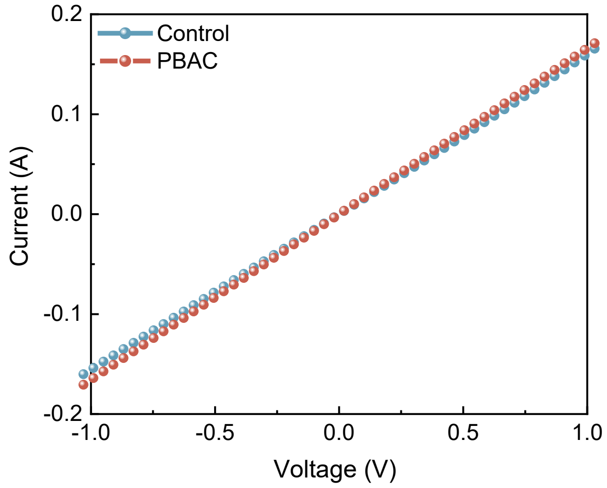

Fig. S9. I-V curves. I-V characteristic curves of the perovskite films without and with PBAC modification.

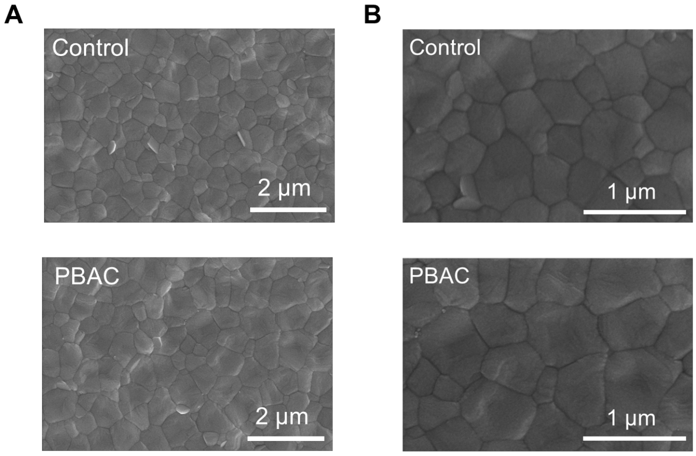

Fig. S10. Morphology of perovskite films. SEM images (**A**) Low resolution, (**B**) High resolution for the perovskite films without and with PBAC modification.

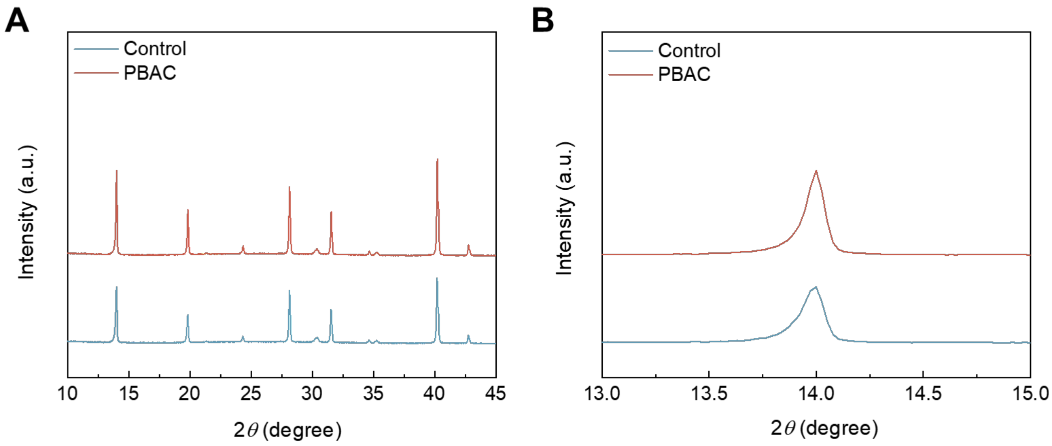

Fig. S11. XRD patterns. (**A**) perovskite films without and with PBAC modification. (**B**) partial enlarged drawing (2θ=13-15°).

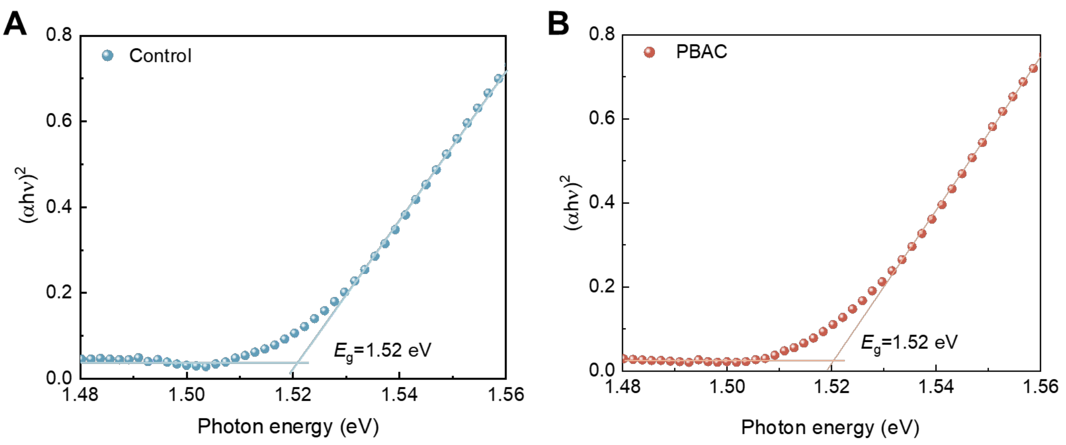

Fig. S12. *T*auc plots of perovskite films. (A) without and (B) with PBAC modification.

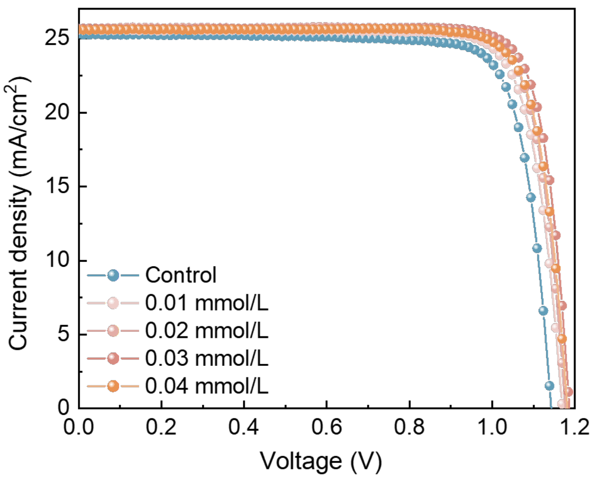

Fig. S13. Devices performance. *J-V* curves of the devices with modification at different concentration.

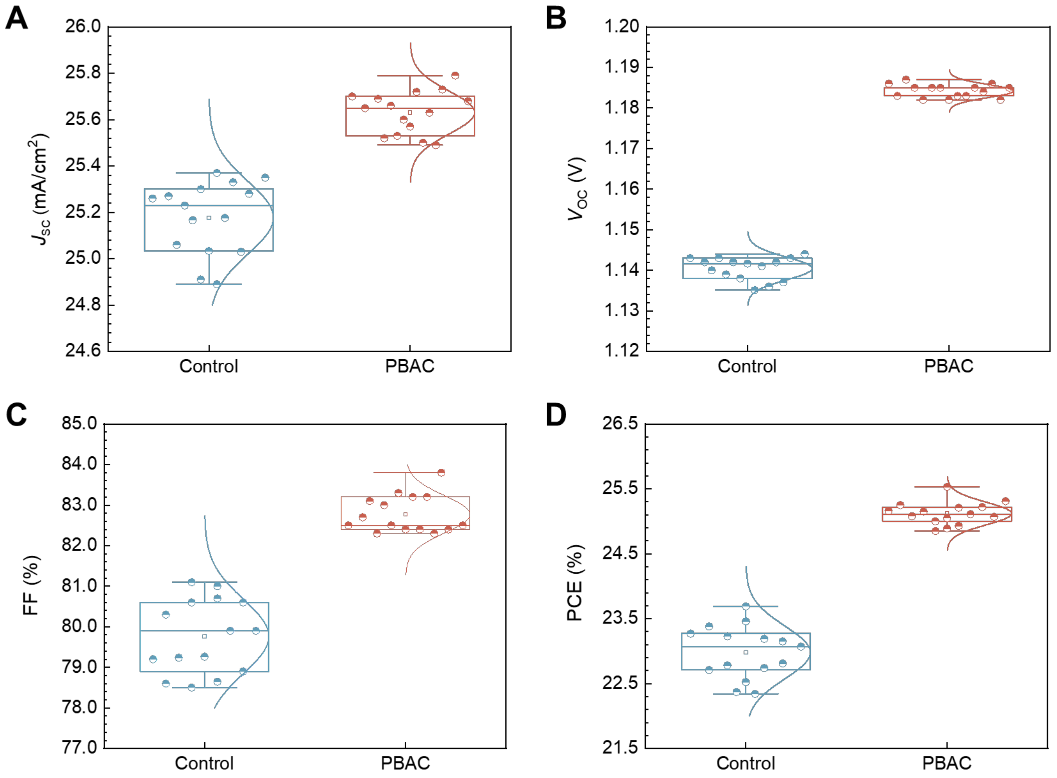

Fig. S14. Statistics of PSCs treated with PBAC, comparing to the control device. (**A**) J_{SC}, (**B**) V_{OC}, (**C**) FF and (**D**) PCE. The statistical data were obtained from 15 individual cells for each kind of device.

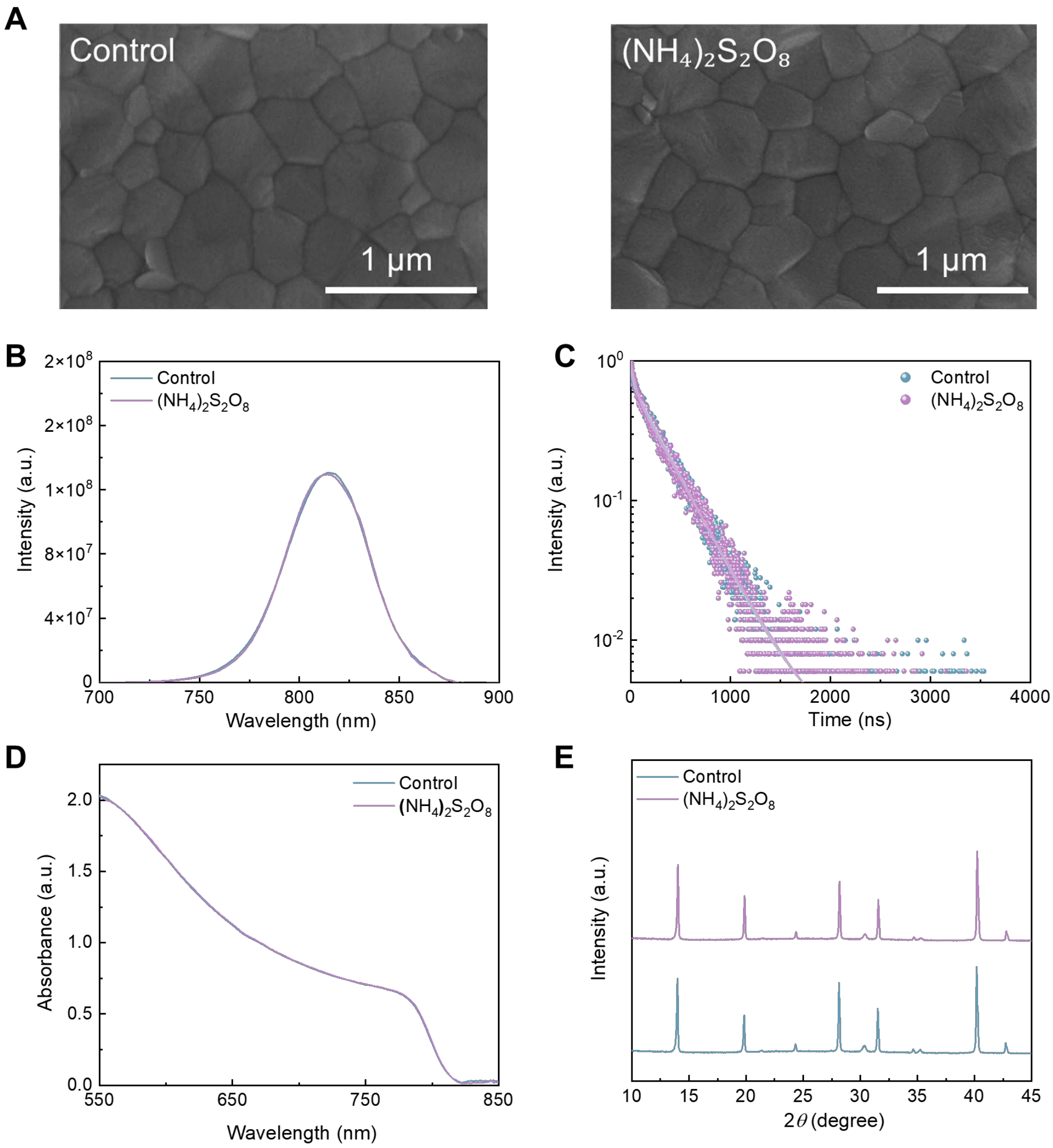

Fig. S15. Effect of $(NH_4)_2S_2O_8$ on perovskite films crystallization. (**A**) SEM images, **(B)** PL, **(C)** TRPL, (**D**) UV-vis spectra and **(E)** XRD of the perovskite films with 1 mol% $(NH_4)_2S_2O_8$ addition relative to PBAC dosage (0.03 mmol/L).

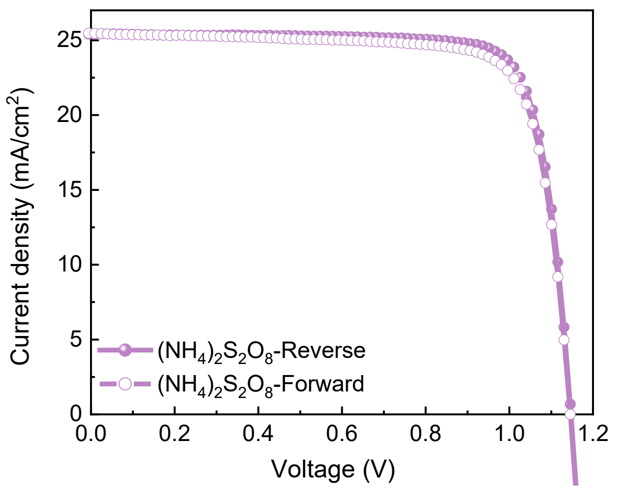

Fig. S16. Devices performance. *J-V* curves of PSCs with $(NH_4)_2S_2O_8$ doping.

中国认可
国际互认
检测
TESTING
CNAS L8490

Test and Calibration Center of New Energy Device and Module,
Shanghai Institute of Microsystem and Information Technology,
Chinese Academy of Sciences (SIMIT)

Measurement Report

Report No. 23TR102501

**Client Name**	Chongqing University
**Client Address**	No. 174 Shazheng Street, Shapingba District, Chongqing
**Sample**	Perovskite Solar Cell
**Manufacturer**	College of Optoelectronic Engineering
**Measurement Date**	25th October, 2023

**Performed by:**	Qiang Shi *Qiangshi*	**Date:** 25/10/2023
**Reviewed by:**	Wenjie Zhao *Wenjie Zhao*	**Date:** 25/10/2023
**Approved by:**	Yucheng Liu *Yucheng Liu*	**Date:** 25/10/2023

Address: No.235 Chengbei Road, Jiading, Shanghai **Post Code:**201800

E-mail: solarcell@mail.sim.ac.cn **Tel:** +86-021-69976921

The measurement report without signature and seal are not valid.
This report shall not be reproduced, except in full, without the approval of SIMIT.

Sample Information

Sample Type	Perovskite solar cell
Serial No.	10-1#
Lab Internal No.	23102501-1#
Measurement Item	I-V characteristic
Measurement Environment	23.9±2.0°C,41.3±5.0%R.H

Measurement of I-V characteristic

**Reference cell**	PVM 1121
**Reference cell Type**	mono-Si, WPVS, calibrated by NREL (Certificate No. ISO 2075)
**Calibration Value/Date of Calibration for Reference cell**	144.53mA/ Feb. 2023
**Measurement Conditions**	Standard Test Condition (STC):<br>Spectral Distribution: AM1.5 according to IEC 60904-3 Ed.3,<br>Irradiance: 1000±50W/m^2, Temperature: 25±2°C
**Measurement Equipment/ Date of Calibration**	AAA Steady State Solar Simulator (YSS-T155-2M) / July.2023<br>IV test system (ADCMT 6246) / June. 2023<br>Measuring Microscope (MF-B2017C) / July.2023<br>SR Measurement system (CEP-25ML-CAS) / April.2023
**Measurement Method**	I-V Measurement:<br>Logarithmic sweep in both directions (Voc to Isc and Isc to Voc) during one flash based on IEC 60904-1:2020.<br>Spectral Mismatch factor was calculated according to IEC 60904-7 and I-V correction according to IEC 60891.
**Measurement Uncertainty**	Area: 1.0%(k=2); Isc: 1.9%(k=2); Voc: 1.0%(k=2);<br>Pmax: 2.4%(k=2); Eff: 2.5%(k=2)

Report No. 23TR102501

====Measurement Results ====

	Forward Scan (Isc to Voc)	Reverse Scan (Voc to Isc)
Area	10.01 mm^2	
Isc	2.557 mA	2.560 mA
Voc	1.179 V	1.179 V
Pmax	2.517 mW	2.526 mW
Ipm	2.438 mA	2.458 mA
Vpm	1.033 V	1.028 V
FF	83.48 %	83.68 %
Eff	25.15 %	25.24 %

- Spectral Mismatch Factor: SMM=0.9950.
- Designated illumination area defined by a thin mask was measured by measuring microscope.
- Test results listed in this measurement report refer exclusively to the mentioned measured sample.
- The results apply only at the time of the test, and do not imply future performance.

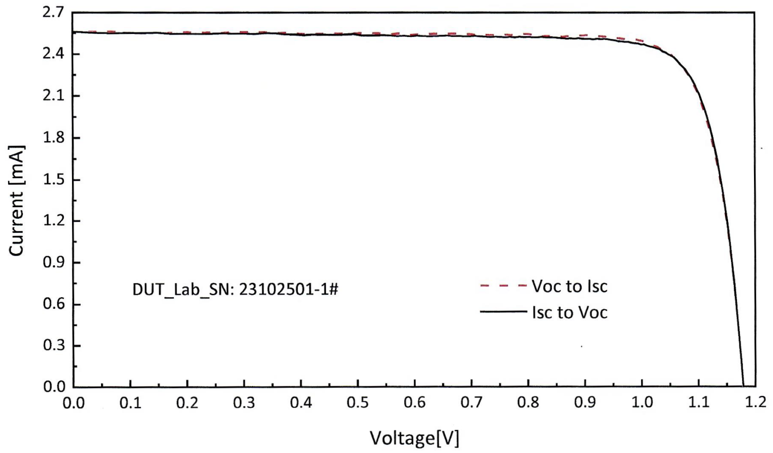

Fig.1 I-V curves of the measured sample

-------End of Report-------

Fig. S17. Certificated results. Independent certification of one of the best-performing target devices by Test and Calibration Center of New Energy Device and Module, Shanghai Institute of Microsystem and Information Technology, Chinese Academy of Sciences (SIMIT).

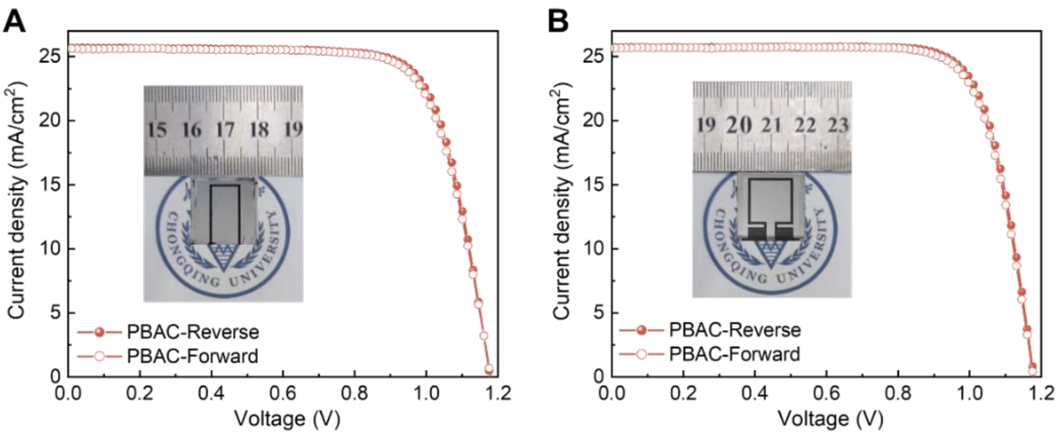

Fig. S18. Devices performance. *J–V* curve of the optimized PBAC modified devices of **(A)** and **(B)** of different rectangle structures with an active area of 1.0 cm^2.

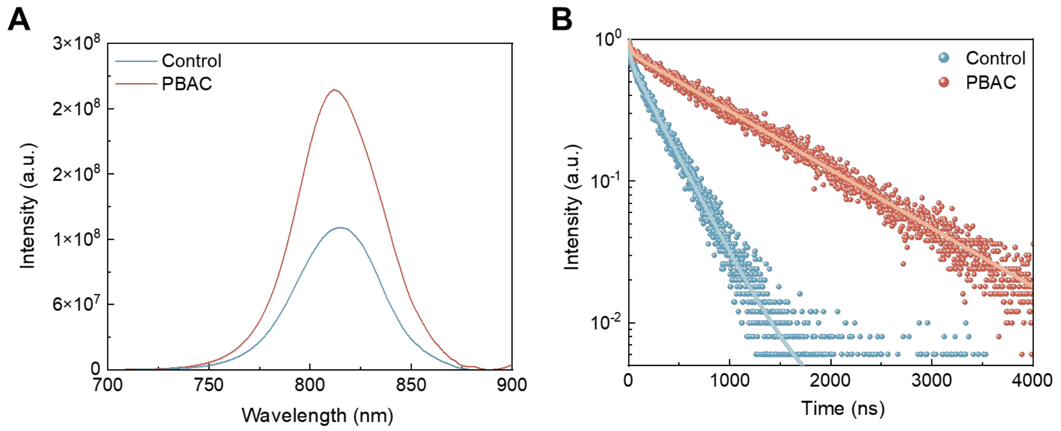

Fig. S19. Carrier dynamics of the perovskite films. (**A**) PL spectra and (**B**) TRPL spectra of the control and PBAC modified perovskite films deposited on glass.

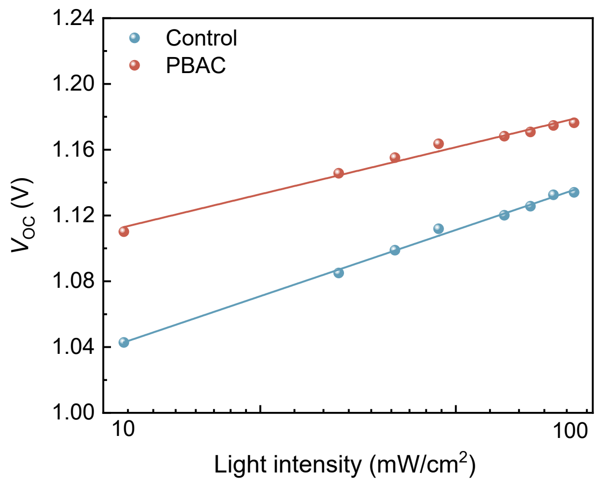

Fig. S20. Ideality factor measurement. Light intensity dependence of the V_{OC} of PSCs.

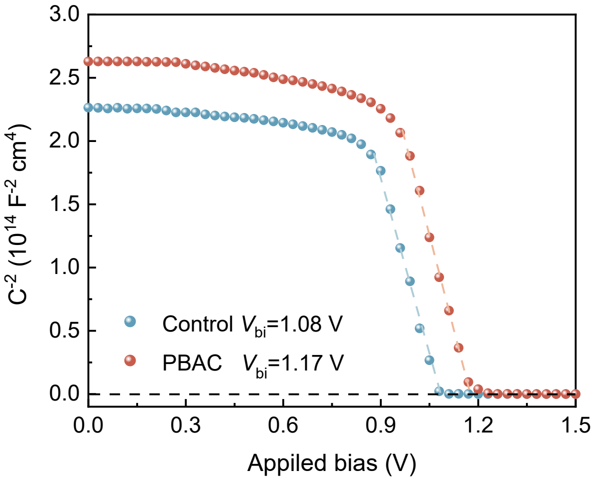

Fig. S21. Mott-Schottky curves. Mott-Schottky curves of the control and PBAC modified devices.

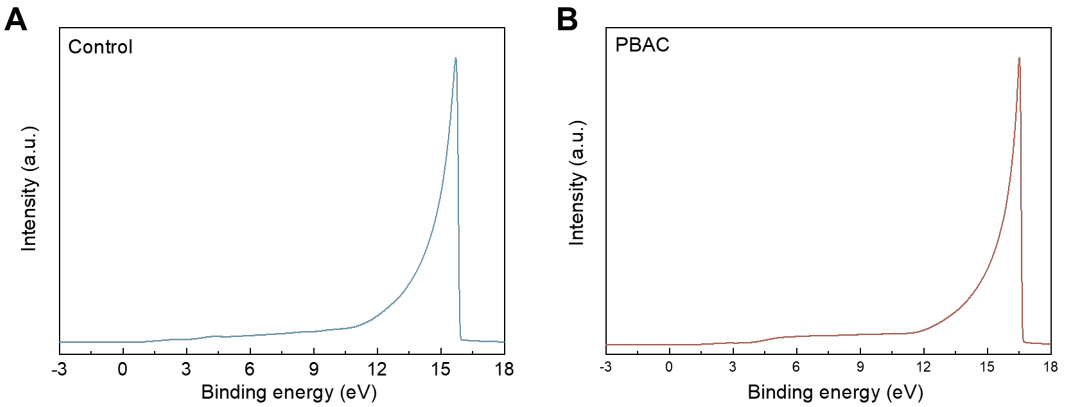

Fig. S22. UPS measurements of the perovskite films (**A**) without and (**B**) with PBAC modification.

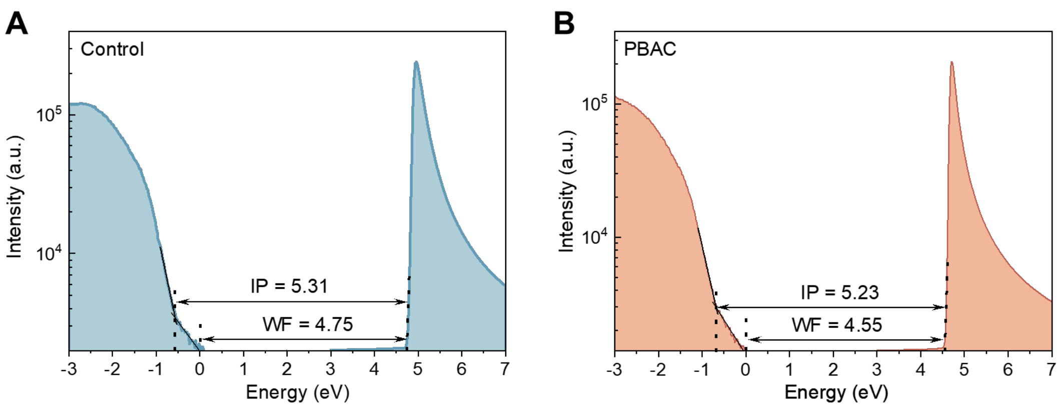

Fig. S23. UPS spectra of secondary electron cutoff region and valence band region of the perovskite films (A) without and **(B)** with PBAC modification. The secondary electron cutoff feature (right peak feature in the panel) is shifted by the incident photon energy (21.22 eV) to show the position of the vacuum level relative to the Fermi level (located at 0 eV), which corresponds to the work function. The valence band maximum (V_{BM}) region (left peak feature in the panel) displays the position of valence band reference to the Fermi level. The combination of the W_F and V_{BM} gives the position of IP.

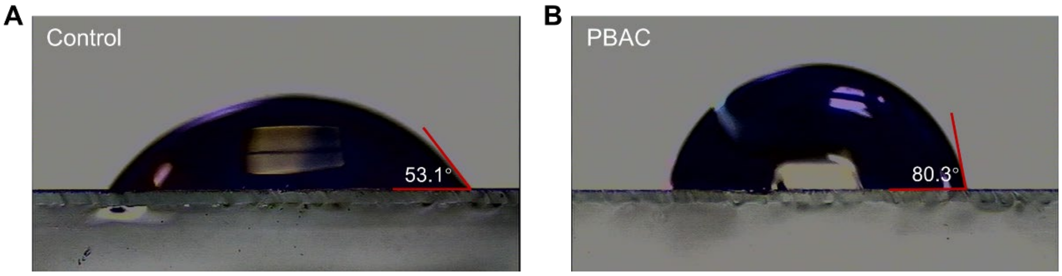

Fig. S24. Water contact angle. (A) control and **(B)** PBAC modified perovskite films.

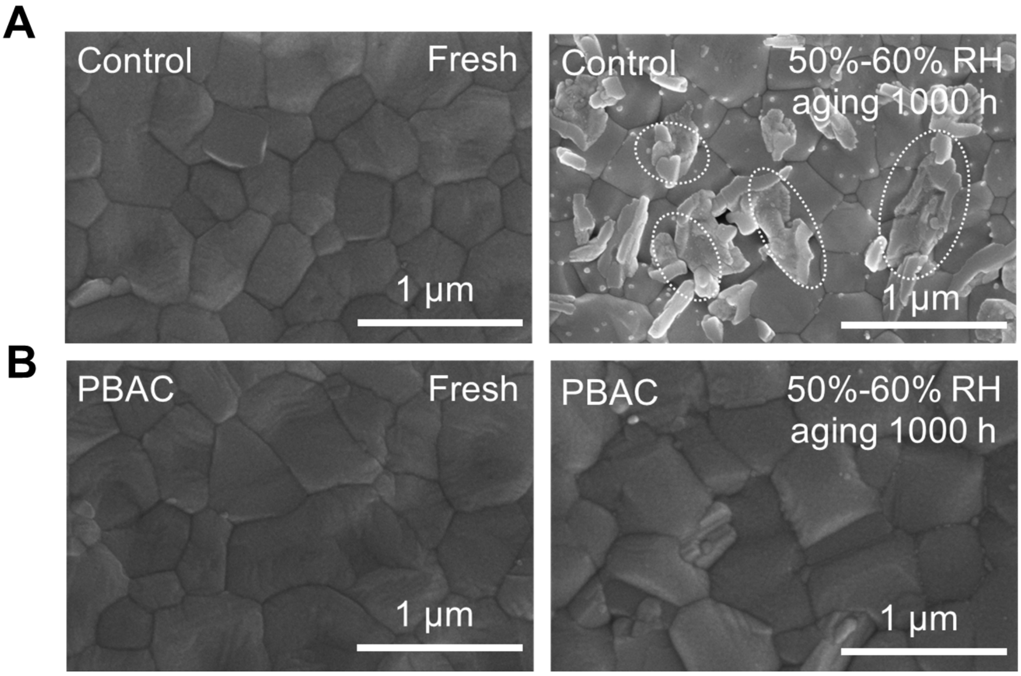

Fig. S25. Morphology of perovskite films. SEM images of perovskite films (**A**) without and (**B**) with PBAC modification before and after aging in 50-60% RH for 1000 hours.

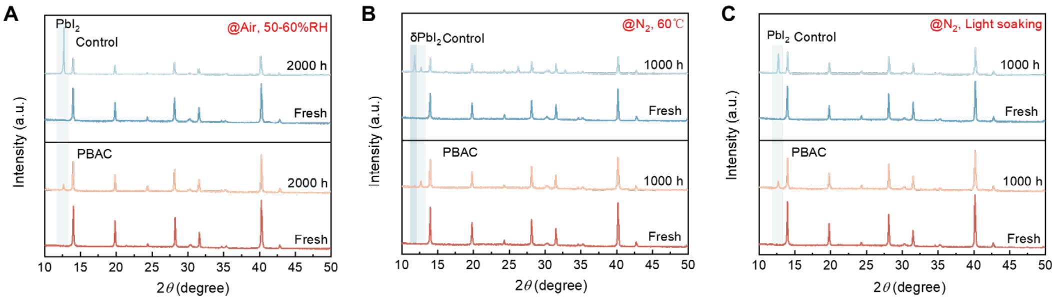

Fig. S26. Effect of the perovskite films stability. XRD patterns of the perovskite films without and with PBAC modification after aging in (**A**) humid air (RH: 50-60%), (**B**) 60°C hotplates and (**C**) one sun light illumination.

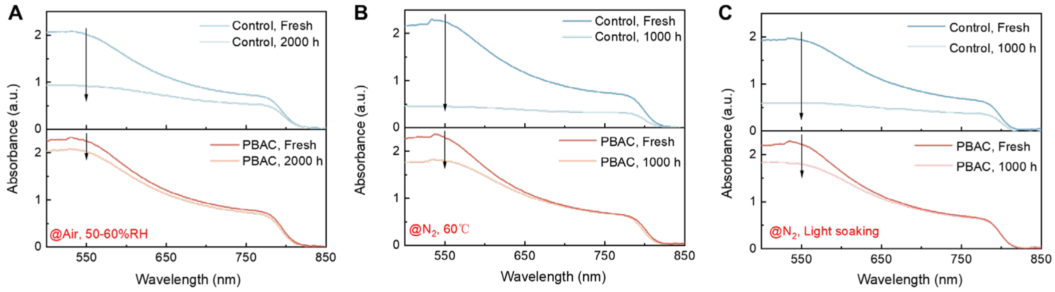

Fig. S27. Effect of the perovskite films stability. UV-vis spectra of the perovskite films without and with PBAC modification after aging in (**A**) humid air (RH: 50~60%), (**B**) 60°C hotplates and (**C**) one sun light illumination.

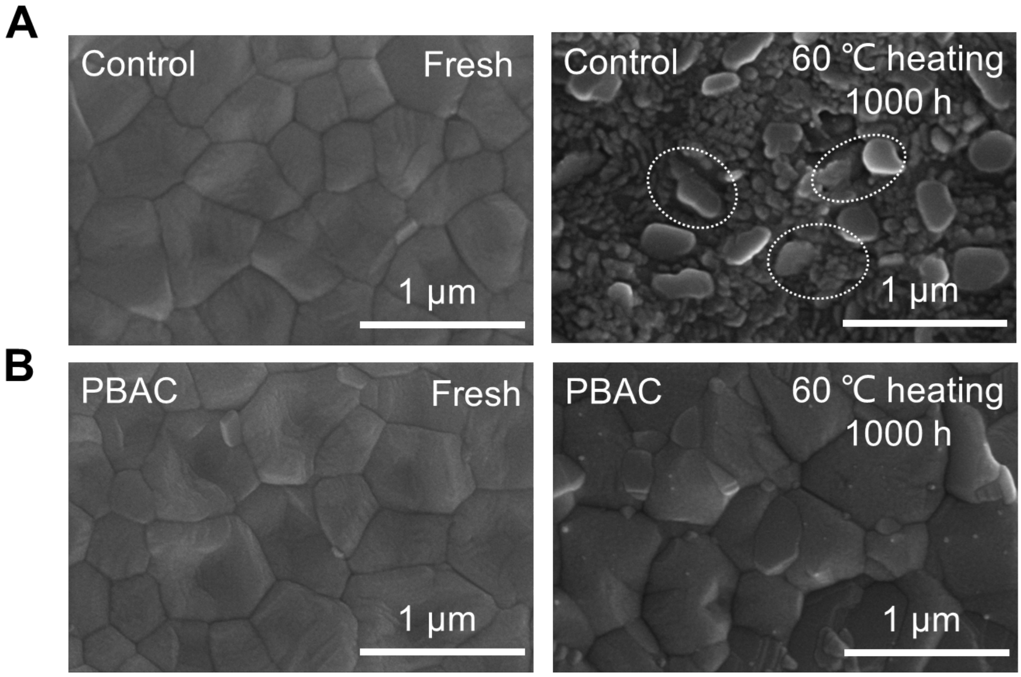

Fig. S28. Morphology of perovskite films. SEM images of perovskite films (**A**) without and (**B**) with PBAC modification before and after aging in 60℃ hotplates for 1000 hours.

2. Supplementary tables

Table S1. Device performance. PV parameters of the champion control and PBAC modified PSCs under AM 1.5G illumination.

Devices	Scan direction	V_{OC} (V)	J_{SC} (mA/cm^2)	FF (%)	PCE (%)
Control	Reverse	1.143	25.31	81.0	23.44
	Forward	1.142	25.23	78.9	22.74
PBAC	Reverse	1.186	25.68	83.8	25.53
	Forward	1.187	25.70	83.3	25.40

Table S2. Device performance. PV parameters of the 1.0 cm^2 champion PSCs with PBAC modification under AM 1.5G illumination.

Device	Scan direction	V_{OC} (V)	J_{SC} (mA/cm^2)	FF (%)	PCE (%)
PBAC	Reverse	1.181	25.81	78.8	24.03
	Forward	1.179	25.62	78.4	23.68

Table S3. Device performance. PV parameters of different rectangle structures of one of the 1.0 cm^2 champion PSCs with PBAC modified under AM 1.5G illumination.

Device	Scan direction	V_{OC} (V)	J_{SC} (mA/cm^2)	FF (%)	PCE (%)
PBAC	Reverse	1.178	25.68	75.7	22.92
	Forward	1.180	25.63	74.8	22.63

Table S4. Device performance. PV parameters of another rectangle structures of 1.0 cm^2 champion PSCs with PBAC modified under AM 1.5G illumination.

Device	Scan direction	V_{OC} (V)	J_{SC} (mA/cm^2)	FF (%)	PCE (%)
PBAC	Reverse	1.180	25.72	77.6	23.56
	Forward	1.178	25.66	76.9	23.23

Table S5. Fitted results of TRPL curves of the perovskite films deposited on the glass substrate without and with PBAC modification.

Samples	Control	PBAC
A_1 (%)	0.29	0.16
τ_1 (ns)	18.15	27.66
A_2 (%)	0.71	0.84
τ_2 (ns)	315.18	1051.54
τ_{ave} (ns)	308.35	1046.44

Table S6. Energy levels of perovskite films extracted from UPS results. Calculated valence band (E_{VB}) and conduction band (E_{CB}) for the perovskite films without and with PBAC modification.

Sample	$E_{F,edge}$(eV)	*W*F (eV)	E_{VB} (eV)	E_g (eV)	E_{CB} (eV)
NiOx			-5.02		-1.17
PTAA			-5.06		-2.14
Control	0.56	-4.75	-5.31	1.52	-3.79
PBAC	0.68	-4.55	-5.23	1.52	-3.71
PCBM			-5.90		-3.90
